# Interaction of SARS-CoV-2 and SARS-CoV-2 vaccines with renin angiotensin aldosterone system, clinical outcomes, and angiotensin (1-7) as a physiological treatment recommendation: hypothesis and theory article

**DOI:** 10.3389/fmed.2025.1612442

**Published:** 2025-07-10

**Authors:** Ali Rıza Aktaş

**Affiliations:** Sincan Training and Research Hospital, Ankara, Türkiye

**Keywords:** SARS-CoV-2 physiopathology, SARS-CoV-2 vaccination, SARS-CoV-2 autoimmunity, long COVID, SARS-CoV-2 treatment, angiotensin (1-7)

## Abstract

Severe acute respiratory syndrome coronavirus 2 (SARS-CoV-2) has affected all of humanity since the first case was reported and spread rapidly around the world, creating a pandemic. Despite the repurposing of many drugs and the development of vaccines, effective treatment and protection are limited. In addition, SARS-CoV-2 continues to be a current public health problem with complications, identifying cases of long-term Covid syndrome, and detection of vaccine-related adverse events. It can be said that the most important factor underlying all these problems is that the interaction between SARS-CoV-2 and renin-angiotensin-aldosterone system (RAAS) is not completely understood despite extensive research. Although different disciplines have limited determinations from their own perspectives regarding the communication with RAAS, it has not been sufficiently expressed in a way to see the whole picture. In this study, it is tried to see the whole picture in the interaction of RAAS and SARS-CoV-2. It is detected inadequacies in treatments and interactions that may be design errors in vaccines. These determinations also show that our templates for producing treatments are not sufficient. For this reason, we have to develop our templates with what we have learned specifically about SARS-CoV-2. Considering the accuracy of our hypothesis on the SARS-CoV-2 - RAAS relationship, Ang(1-7) can be considered a strong option for treatment. Although the SARS-CoV-2 pandemic seems to be over, epidemics and even new pandemics are likely to occur with new mutations.

## 1 Introduction

A major problem in the pathogenesis of severe acute respiratory syndrome coronavirus 2 (SARS-CoV-2) infection is that angiotensin-converting enzyme 2 (ACE2), within the renin-angiotensin-aldosterone system (RAAS), fails to fulfill its physiological roles following viral interaction (1–5). It can be said that overlooking the role of ACE2 in physiopathology poses significant challenges in designing vaccines and treating SARS-CoV-2 infection. Therefore, it is crucial to consider the negative consequences of existing vaccines and treatment protocols on ACE2 interactions and to develop new therapeutic strategies to address these issues. In this study, the use of angiotensin Ang(1-7) was considered as a new therapeutic approach, supported by a review of literature highlighting its significant role in the circulatory system (6, 7).

### 1.1 Background

Our experience suggests that developing a vaccine should be prioritized to protect the population during the SARS-CoV-2 pandemic (8, 9). The S1 portion of the spike (S) protein of SARS-CoV-2 plays a critical role in binding to ACE2 and facilitating viral entry into the cell (10, 11). S1 has been targeted in the design of vector, protein and mRNA (messenger ribonucleic acid) vaccines (12, 13). Attenuated virus vaccines, vector vaccines, protein and mRNA vaccines have been deployed globally (14, 15). It is hypothesized that recombinantly produced ACE2 could be used for neutralization purposes to prevent the virus from entering cells, and experiments were conducted to this end (16, 17). Monoclonal antibodies (MAbs) produced against S1 have been used for neutralization (18, 19). Antivirals that inhibit RNA-dependent RNA polymerase have been employed (20–22). Plasma from individuals immune to SARS-CoV-2, along with methylprednisolone, dexamethasone, and hydrocortisone, has been used in coronavirus disease (COVID-19) treatment (23–27). Additionally, chloroquine and colchicine were also utilized (22). Oxygen replacement therapies were administered to stabilize patients (28, 29). MAbs, targeting cytokines, such as interleukin (IL) 1 and IL6, were used to mitigate the effects of heightened cytokine activity and levels in clinical scenarios following SARS-CoV-2 infection (26).

Subsequent to these treatments and vaccinations, some complications and inadequacies were observed in studies monitoring the pandemic’s progression, which were subsequently published and shared (27–34). New SARS-CoV-2 variants were detected, becoming dominant in various periods and regions during the pandemic (11, 14). These variants sparked new epidemic (14, 15) waves and raised fresh concerns about ongoing pandemic conditions. Additionally, a syndrome known as “long-term COVID” was identified following SARS-CoV-2 infection and vaccination (35–38).

In this study, the following sequential questions, implications and possible solutions arising from the interaction between SARS-CoV-2 and RAAS were identified.

(1) As a result of the interaction between SARS-CoV-2 and ACE2, ACE2 cannot function. ACE2 catalyzes Ang2 to form Ang(1-7). What happens when Ang2 increases in tissues and circulation because Ang2 cannot be catalyzed when the ACE2 enzyme interacts with SARS-CoV-2?
(2) As a result of ACE2’s inability to form Ang(1-7), Ang(1-7) deficiency occurs. What are the consequences of this situation in cells and tissues?
(3) ACE2 is a member of RAAS. What is the role of RAAS? (39)
(4) Can perfusion be impaired due to a disorder in the RAAS and can hypoxia occur in different tissues due to impaired perfusion?
(5) What are the clinical symptoms and abnormal laboratory findings that occur when hypoxia occurs in tissues and cells due to impaired perfusion? How do they correlate with the symptoms and abnormal laboratory findings in SARS-CoV-2 infection?
(6) What is the reaction of the immune system in the acute, subacute and long term when there is tissue and cell damage due to hypoxia?
(7) Do adaptive components of the immune system cause autoimmune reactions when they come into play in hypoxia-related cell and tissue loss? How does it correlate with findings in SARS-CoV-2 infection?
(8) Since SARS-CoV-2 enters cells by binding to ACE2 with the S1 protein (39), shouldn’t S1 have similar epitopes to Ang2, the natural substrate of ACE2?
(9) If Ang2 and S1 have similar epitopes, is there a cross-reactivity to Ang2 after SARS-CoV-2 infection and immunization with the S1 component in vaccines (29)?
(10) Can the attenuated SARS-CoV-2 virus used in vaccines bind to ACE2? If this complex enters the cell (7), can ACE2 still perform its function in the RAAS?
(11) Since mRNA vaccines target immunization by inducing cells to produce the S1 protein, which facilitates SARS-CoV-2 entry into the cell (16, 40–42), it is likely that these S1 proteins bind to ACE2. In this case, can ACE2 perform its normal function in the RAAS?
(12) Given that Ang2 interacts with AT1 and AT2 receptors beyond the ACE2 enzyme (43–47) and the structural similarities between S1 and Ang2, could S1 also interact with AT1 and AT2 receptors? Has this potential interaction been confirmed, and if so, what are the implications?
(13) Why are we advocating a new treatment? How and why was this particular approach chosen?

To address these questions, we will first review SARS-CoV-2 phylogenetics and morphology, then histopathological and pathophysiological findings in SARS-CoV-2 infection and then present scenarios to facilitate discussion and deepen our understanding of these issues.

### 1.2 Hypothesis

Approximately 5 years have passed since the beginning of the SARS-CoV-2 pandemic. During this period, it can be stated that adequate treatment and protection could not be provided in the light of the information obtained regarding the symptoms and laboratory findings regarding acute, subacute and long covid syndrome related to SARS-CoV-2 complications. With this acceptance and justifications, I have reached concrete and abstract conclusions regarding the necessity of re-questioning the pathophysiology in the context of SARS-CoV-2 - ACE2 - RAAS. These hypotheses based on the results I obtained are as follows:

(1) There are adverse cause-effect relationships between acute, subacute and PASC in the SARS-CoV-2 - ACE2 - RAAS and SARS-CoV-2 - ACE2 - Vaccine interaction.
(2) Considering the design flaws in current vaccines and the inadequacy of treatments, new vaccines and treatments need to be developed.
(3) Ang(1-7), which has been studied before, has the potential to eliminate the currently identified problems.

## 2 Methods

For this study on SARS-CoV-2, the Pubmed, the ResearchGate and IOMC databases, which include all open access article formats published in English, was searched with the keywords “SARS-CoV-2” and “COVID-19” for a period of approximately 5 years from the end of December 2019 to October 2024.

In order to clarify the data and information obtained with the keywords “SARS-CoV-2” and “COVID-19” in the scan and to establish the mechanisms and connections that form the whole picture, the scan is developed and expanded.

For this purpose, new keywords are SARS-CoV-2 morphology, phylogenetics, transmission route, cells and tissues to which the virus shows tropism, replication stages, SARS-CoV-2’s relationship with ACE2, the fate of ACE2, the role of ACE2 in RAAS, the role of RAAS in the body, the role of RAAS in ventilation and perfusion, the relationship of RAAS with acute respiratory distress syndrome (ARDS), hypoxia in cells and tissues as a result of perfusion disorder, cell response to hypoxia, the relationship of this response with cytokine storm, the effect of hypoxia and cell death on the immune system, organ involvement, clinic, prognosis, conditions affecting prognosis, drugs used for treatment and their contribution to the treatment during the process, tolerance, side effects and limitations of vaccines, mechanisms of immunization against SARS-CoV-2 and vaccines created, components of SARS-CoV-2 vaccines. The search was expanded to examine the theoretical possible interaction of SARS-CoV-2 S1 with RAAS.

**FIGURE 1:**
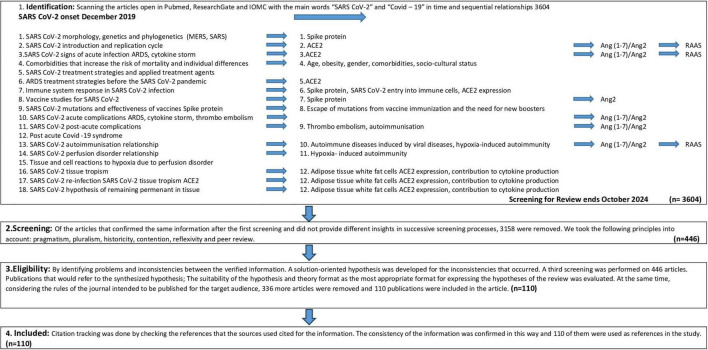
Flow diagram illustrating the screening and selection process.

After collecting the information pool, the intersection points detected in the articles were identified in order to see the whole systemic picture. These intersections were determined as SARS-CoV-2 S1, ACE2, RAAS, Ang 2, Ang(1-7), ARDS, ventilation disorder, perfusion disorder, cell and tissue damage, autoimmunity and post-covid syndrome. These points were scanned for the second time and the hypothesis were clarified with these articles. These intersection concepts and assumptions were selected in a way that would not accept any opposition. For example, the relationship between SARS-CoV-2 and S1 and ACE2, such as ACE2 being an element of RAAS, were aimed to prevent bias and strengthen the synthesis of the hypothesis. Due to the reference limit for the reviews, 113 of them were selected for reference that would confirm the data and information in this synthesis. This process is schematically shown in Figure 1.

In order to see the whole picture, the symptoms, current connections and results in all systems were examined. When the role of perfusion disorder in the main pathology was understood, the RAAS-SARS-CoV-2 relationship was tried to be explained by revealing the sample pathologies on some systems. Since the systematic review meta-analysis format is suitable for working in a relatively narrow area and is not suitable for showing the whole picture, the article was presented in the narrative review format. In this way, multidisciplinary data could be hypothesized as a whole.

## 3 Results and discussion

### 3.1 SARS-CoV-2 evolution and morphology

The Coronaviruses (CoV) contain big envelope, not segmented, one-chained, positive RNA. Their genoms are 27–32 kb (19, 48). CoVs are classified in four groups: α, β, γ, δ. From αCoVs, HCoV-229E and HCoV-NL63, from βCoVs, HCoV-HKU1 and HCoV-OC43 make respiratory infection like mild cold (49, 50).

Severe acute respiratory syndrome coronavirus, MERS CoV (50, 51) and SARS-CoV-2 of βCoV make respiratory infection (10, 52). The gene analysis of SARS-CoV-2 is made and found to be similar to bat CoVRaT G13 with 96.2%. Its similarity with SARS-CoV is found to be 79.5%. As a result of these studies, it is doubted that the host of SARS-CoV2 could be the bat. It is certain that SARS-CoV-2 and SARS-CoV enter with ACE2 receptor on the host cell in human (53, 54). The CoV contains 4 structural proteins. These are S (spike), envelope (E), M (membrane) and N (Nucleocapsid) (52, 55).

Spike protein (S protein) contains S1 and S2 sub-unites. There is Receptor-Binding Domain (RBD) in S1 unit which connects to host cell. The S2 unit helps interaction of virus envelope with the host cell membrane (50, 52). Both SARS-CoV-2 and SARS-CoV show cross reaction in serological tests (56). They have similar series on RBD (16, 52). It is showed that the affinity of the SARS-CoV-2 to ACE2 receptor is 10–20 times higher than that of SARS-CoV (56, 57). There are RBM (Receptor Binding Motif) regions in the RBD (53).

The SARS-CoV-2 spike protein as well as isolated spike- proteins downregulate ACE2 (50). In SARS-CoV infection, the cholesterol in the targeted cell plasma membran plays an important role. It is observed that the interaction of S proteins of the virus with ACE2 in cholesterol-rich regions in the host cell membrane is stronger. The attachment rate of soluble S protein in decreased-cholesterol cell which makes expression of ACE2 decreased by 50% (54). It is showed that ACE2 expression does not decrease in depleted cholesterol cells. The virus attachment can only be with multivalent hold. It is asserted that the cholesterol in the plasma membrane can be decreased with the medicines such as methyl–β-siklo dekstrin (mβCD = methyl-β-cyclodextrin) and a decrease in virus infection can be observed (54).

Severe acute respiratory syndrome coronavirus Spike glycoprotein is a class 1 viral fusion protein. In all coronaviruses, the part of proximal transmembrane which is rich of aromatic amino acid of S protein, Juxtamembrane domein (JMD), is highly protected. Those aromatic amino acids in the JMD in the SARS S glycoprotein play a critical role in virus-cell, and cell-cell fusion.

They include 2 series of 7 repeating amino acid, which are called as HR-1 and HR-2. They are hydrophobic fusion peptits. The P*^H^* is a trigger for connecting to receptor. After this triggering, interim confirmation and lastly after fusion 6 helix bundle confirmation can happen. The HR2 C terminal homologous peptits can inhibit the entry of coronavirus into the cell. It is shown that against this region the virus entry can be blocked with a FIV (feline immunodeficiency virus) monoclonal antibody and with a JMD-like oktapeptit (48).

The coronavirus is developed by budding from the endoplasmic-golgi region. The coronavirus envelopes contain ≈8% semilysobisphophatidic acid (SLBPA), ≈40% phosphatidylcholine, ≈10% sphingomyelin and ≈12% phosphatidylinositol. SLBPA is not included in plasma membrane but it is SLBPA-rich in golgi region (48). It is thought that the lipid structures of the coronaviruses may have a contribution in fusion of the host cell with plasma membrane.

### 3.2 Histopathological findings in SARS-CoV-2 infection

The histopathological finding seen in 86% of patients who succumbed to SARS-CoV-2 infection, involving lung edema, prominence of type 2 pneumocytes, and prominence of intra-alveolar fibrin and hyaline membranes, is diffuse alveolar damage (22). Interstitial fibrosis and myocyte hypertrophy in the heart were noted in all cases examined (100%). Additionally, post-mortem pathological examinations revealed replacement fibrosis, myocardial amyloid deposits, and myocarditis (58).

In a separate study a major pathological thrombotic event was noted in 100% of the patients, affecting at least one major organ (22). The most affected organs were the lungs (89%), heart (56%), and kidneys (44%). Additionally, diffuse alveolar damage was the most frequent histopathological finding in this cohort, consistent with earlier observations (59). Pathophysiology of SARS-CoV-2 infection; coagulation abnormalities and disruption of factors secreted by endothelial cells create a thrombotic condition in blood vessels. Therefore, early functional impairment of endothelial cells, which can be found shortly after SARS-CoV-2 infection, represents the main pathology of SARS-CoV-2 disease state. It is responsible for systemic vascular dysfunction in terms of hospitalization and death accompanying the disease. In particular, the molecular interaction of SARS-CoV-2 with the ACE2 receptor on the endothelial cell surface at the pulmonary and systemic levels leads to early deterioration of endothelial function, which is followed by vascular inflammation and thrombosis of peripheral blood vessels (60).

### 3.3 SARS-CoV-2 physiopathology and immunopathology

S1 of SARS-CoV-2 and S1 produced by vaccines form a complex with ACE2 and enter cells through this interaction (61, 62). If the host cells are immune cells, S1 is internalized, and it is mostly presented to T helper (T4) and B memory lymphocytes by dendritic cells (44). These T4 and B memory lymphocytes transform B lymphocytes into plasma cells. It also organizes the formation of specific T cytotoxic lymphocytes. With the specific antibodies and T cytotoxic lymphocytes produced by plasma cells, adaptive immunity is formed against S1 of SARS-CoV-2 and S1 of the vaccine.

For S1 to bind ACE2, it is demonstrated that there is an epitope homology between S1 and Ang2, a substrate of ACE2 (29, 47). The ACE2 enzyme converts Ang2 into Ang(1-7) (47, 58, 63). Ang 2 is converted from Ang 1 to Ang II, an 8 aa active peptide, by ACE (64). Immunization against the S1 protein can produce antibodies that may cross-react with Ang2, leading to a decrease in Ang2 levels (29). Ang2 is the main active component of the RAAS with its potent vasoconstrictive, sodium-sparing, proinflammatory and profibrotic effects. Ang(1–7) exhibits vasodilatory, antiproliferative, anticoagulant and antifibrotic activities through the Mas receptor (MasR), thus counterbalancing the negative effects of Ang 2 mediated by AT1R.

Given the possibility of structural similarities between S1 and Ang2 epitopes (29, 47, 61), it is conceivable that S1 might interact not only with ACE2 but also with Ang2 receptors, AT1 and AT2 (46, 64). This interaction could potentially impair the functional roles of AT1 and AT2 receptors.

If AT1, AT2, and ACE2 receptors, which normally interact with Ang2, are blocked by S1, the biological effects typically mediated by Ang2-AT1, Ang2-AT2, and ACE2-Ang2 interactions may be inhibited. To compensate, ACE activity might increase to convert Ang1 into Ang2. ACE2 may also enhance expression on cell surfaces for two reasons: to compensate for reduced Ang2 and to facilitate the conversion of Ang2 to Ang(1-7). ACE, being a primary enzyme that catalyzes the conversion of Ang(1-7) to inactive metabolite in the lungs (6), could exacerbate conditions, such as ARDS (65, 66). A study involving the SARS-CoV virus, which similarly binds to ACE2 and shares some characteristics with SARS-CoV-2 (5, 10), demonstrated a 10-fold increase in ACE2 expression on the apical surfaces of cells (47).

To maintain perfusion, the body requires a balance between vasoconstriction and vasodilation, influenced by both time and localization (6, 60, 67). Ang2 and Ang(1-7) play crucial roles in this process, exerting opposing effects to regulate perfusion (6, 7, 67). However, SARS-CoV-2 interaction with the ACE2 receptor disrupts this balance (68, 69), impairing necessary organ perfusion at multiple sites and different times. So far, numerous reports have linked the devastating organ injuries to viral homing and attachment to organ-specific cells widely expressing ACE2 (70, 71). In the studies conducted after COVID-19, findings of pathology and multi-organ failure in many organs have been expressed (32–36, 71). To counteract this imbalance, compensation mechanisms (72) are activated. Cytokines, interferons, and hormones are released for intracellular and extracellular interactions (73, 74). Consequently, cell surface receptors and enzymes involved in these interactions may either increase or decrease (75, 76), leading to elevated cytokine levels in the blood of patients with SARS-CoV-2 infection (77, 78).

These variations in cytokine levels can be attributed to the different renewal and proliferation rates of cells and tissues (73), allowing cytokines to enter the general circulation at varying times (74, 77). We also know that Arachidonic acid is converted into thromboxane A2, a powerful pro-aggregation and vasoconstrictive factor. Thromboxane A2 activates platelets and contributes to thrombo embolism by providing their aggregation (66). The source of this Arachidonic acid may be the increased fat tissue in people with increased incidence of SARS-CoV-2 mortality due to obesity, diabetes and cardiovascular diseases. ACE2 expression has a significant expression in fat cells. Moreover, ACE2 expression is more intense in white fat cells, which are dense in this mass, and brown fat tissue is more abundant in infants and Eskimos. This may explain some of the increased mortality rates in these individuals. In addition, there are publications before COVID-19 that have detected increased cytokine levels *in vitro* in this fat tissue due to polarized macrophages and lipopolysaccharides (66).

Furthermore, hemodynamic disturbances caused by SARS-CoV-2 infection contribute to systemic hypoxia (79–81), activating cellular responses that eventually lead to cell death and disrupted cell integrity (32, 81). Moreover, SARS-CoV-2 infection-induced hypoxia increases blood viscosity, contributing to thrombosis in blood microvasculature (66). Previous studies on primary cultures of type II alveolar epithelial cells have shown that Ang2 induces apoptosis *in vitro* in a dose-dependent manner at 10 μmol/L Ang2 (66). This may be considered for blood vessel endothelial cells that express ACE2 and reach every tissue.

Exposed cell components are then recognized by the immune system (32, 82), triggering an adaptive response and leading to autoimmunization against these components (83–85). This process can cause an overactive immune response, with immune cells migrating from the circulation to lymphoid tissues or areas where integrity is compromised (80, 82), and sometimes disappearing (79). This scenario also includes the development of autoantibodies against cell components, such as ACE2 and ACE, as noted in some studies (30). Accepting this perfusion defect in the pathophysiology of SARS-CoV-2 allows for the detection of hypoxia-induced changes and autoantibodies against other cellular components (86–88), potentially explaining the mechanism behind post-COVID autoimmune diseases.

**FIGURE 2:**
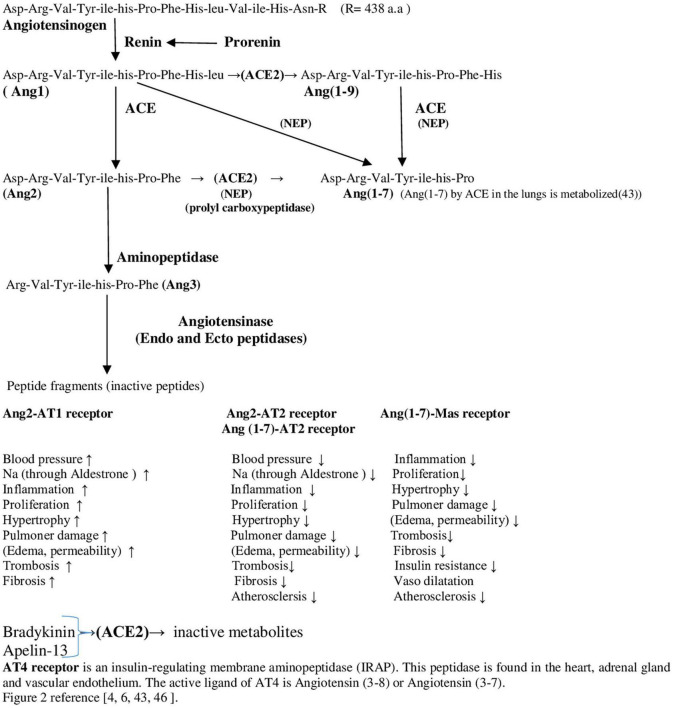
Renin angiotensin system flow chart.

After SARS-CoV-2 infection and vaccination, S1 may react with AT1, and AT2 (29, 89–91). This situation may disrupt the functions performed by Ang2 through its combination with AT1 and AT2. This disruption affects both the necessary vasoconstriction and vasodilation for proper perfusion (6, 10). Specifically, Ang(1-7) production from Ang2 by ACE2 is inhibited by the S1 peptide used in vaccines and by S1 from SARS-CoV-2 during (92) infection. This contributes to vascular blood pressure dysregulation observed as both hypertension (93) and hypotension, and can also lead to myocardial ischemia, arrhythmias, vegetations, and an increased risk of thromboembolism (36–38). The cumulative effects of excessive Ang2 and deficient Ang(1-7) further clarify the situation (43–45) (Figure 2).

Furthermore, the potential of S1 from mRNA vaccines to bind to AT1 and AT2 receptors and affect their function in the RAAS warrants further investigation and clarification (12, 29). This research could provide insights into the pathogenesis of SARS-CoV-2 and post-vaccination complications.

### 3.4 Scenarios of reaction between S1 and Ang2

Based on the hypothesis presented, we can analyse the lung pathologies most severely affected by SARS-CoV-2, which contribute to mortality, by exploring different scenarios of interaction between S1 of the virus and Ang2.

*Scenario 1: No cross-reactivity between S1 and Ang2*

Assuming that there is no cross-reactivity between S1 and Ang2 in the lungs, the blockade of ACE2 by S1 prevents Ang2 from interacting with ACE2, leading to Ang2 accumulation and Ang(1-7) reduction.

*Scenario 2: Cross-reactivity between S1 and Ang2 post-adaptive immunization*

In this scenario, after a period following SARS-CoV-2 infection, there is a cross-reaction between S1 and Ang2. This interaction decreases both Ang2-AT1 and Ang(1-7)-Mas receptor interactions. The resultant disruption in perfusion and vascular endothelial integrity leads to fluid accumulation and retention in the lung parenchyma, migration of inflammatory cells to the parenchyma, and impaired ventilation, culminating in ARDS (79, 81). The accompanying hypoxia from impaired perfusion in other organs leads to cellular breakdown and death (94–98). The immune system engages to clear the cellular debris, and the adaptive immune response promotes the formation of cytotoxic T lymphocytes and antibody-producing plasma cells in increased numbers and diversity (91, 94). As the body’s compensation mechanisms intensively activate against ongoing perfusion disruptions, a severe cytokine storm ensues. This systemic response impairs perfusion in other tissues, leading to multiple organ failure (71). Under these severe conditions, treatment becomes increasingly challenging, potentially resulting in patient mortality (60, 99).

#### 3.4.1 Importance of ACE2 and role of Ang(1-7) in these scenarios

The ACE2 enzyme is an important member of the RAAS (7, 69). The RAAS is a biochemical system with an active cascade (Figure 2) (6). The RAAS regulates cell growth and proliferation, inflammation, and cytokine formation (6, 47). It has paracrine, autocrine, and intracrine effects on local tissues (6). Apart from in the lungs, ACE2 is predominantly expressed in the heart; kidneys; testicles; endothelial cells; and smooth muscle cells in many tissues, such as blood vessels, intestines, pancreas, and adipose tissues (93, 97). Therefore, the areas where the immune system fights SARS-CoV-2 are all cells and organs that express ACE2.

*What type of a protein is ACE2?*

Angiotensin-converting enzyme 2 is a chimeric protein formed by the combination of two genes (47, 94). ACE2 is a mono carboxy peptidase containing 805 aa (amino acids) (5, 47). ACE2 contains a single catalytic domain (4). The extracellular catalytic portion of ACE2 is 147–555 aa (43). The substrate-binding part is 273–345 aa, and the region in the plasma membrane (transmembrane) is 740–768 aa (43, 96).

Its catalytic part, which is a metallopeptidase that uses histidines and Zn (zinc) ions, is between 374–378 aa (47, 97). The HEXXH (His-Glu-Xaa-Xaa-His) motif is the active part of ACE2 (47, 98). In non-covalent bonds, a water molecule is used in the intermediate reaction. This substrate facilitates the nucleophilic attack on carbonyl bond (47). The hinge bending action of native ACE2 creates an on-off switch in the catalytic infrastructure (47, 99). With the new position of the key fragment for catalysis, Ang(1-7) is formed by the removal of the last amino acid, phenylalanine, at position 8 from Ang2 by ACE2 (16, 44, 64).

*What are the roles of ACE2 in other systems, and what happens when it fails to function against SARS- CoV-2 pathogenesis?*

Angiotensin-converting enzyme 2 hydrolyses peptides, such as apelin, opioids, and quinine (6). Apelin is known to play an important role in myocardial contraction and blood pressure regulation (6, 47). ACE2 also stabilizes neutral amino acid carriers and is implicated in the pathology of Hartnup disease (1, 43, 47). Furthermore, ACE2 regulates the digestive system microbiome by controlling diarrhea and inflammation (1, 43). The non-catalytic domain of ACE2 has been shown to be important for amino acid reabsorption in the kidney and pancreatic beta-cell proliferation (1, 47).

The human ACE2 gene is located on the X chromosome (4, 6), and ACE2 levels in circulation are higher in men than in women (57, 75). This difference may partially explain the higher mortality rates observed in male patients with SARS-CoV-2 infection. Studies on genetic analysis of ACE2 polymorphisms may partially explain the different mortality rates of COVID-19 disease (39). ACE2 levels decrease with age, a reduction associated with cardiomyopathy, as well as kidney and lung diseases (4, 55). In elderly patients, ACE2 deficiency leads to an increased Ang2-AT1 receptor effect, reduced Ang(1-7) production, and a greater orientation of Ang2 toward AT1 receptors, all contributing to higher mortality rates (55, 75).

Additionally, some studies suggest that immune system cells express little or no ACE2, a phenomenon that warrants further investigation to clarify its mechanisms (61, 62). TMPRSS2 (Transmembrane protease, serine 2) plays a critical role in facilitating SARS-CoV-2 entry by interacting with the S2 region of the viral S protein (11, 39, 93). TMPRSS2 and Human Airway Trypsin (HAT), expressed by ACE2-positive cells in the human lung (50), cleave ACE2 by targeting arginine and lysine residues at positions 697 and 716, respectively, enabling SARS-CoV-2 S protein cleavage and increasing viral entry (50).

Evidence suggests that ACE2 interaction with SARS-CoV is enhanced by ADAM17 (ACE2 disintegrin and metallopeptidase 17) and TACE (tumor necrosis factor-α converting enzyme) (50). TMPRSS2 inhibitors, such as camostat mesylate, have been explored as treatments to block SARS-CoV-2 entry into host cells (50, 100).

Recombinant human ACE2 (rhACE2) has been shown to suppress oxidative stress induced by Ang2, reduce the expression of profibrotic genes, and inhibit ERK1/2 signaling through Ang(1-7) in heart muscle cells and fibroblast cultures (44). ACE2 exerts therapeutic effects on the heart, kidneys, and lungs by counteracting the detrimental effects of Ang2 mediated by ACE (4, 43). Additionally, recombinant ACE2 has been used in patients with ARDS of various etiologies, where it has contributed to recovery (4, 16). Infusion of recombinant ACE2 in patients with pulmonary hypertension has been shown to decrease oxidative and inflammatory markers while improving pulmonary hemodynamic (47, 79).

Animal studies indicate that AT1 receptor blockers (ARBs) increase membrane-bound ACE2 by elevating Ang2 levels (7, 100). However, the doses of ARBs required to increase ACE2 expression are higher than those used for hypertension treatment (3, 100).

Angiotensin-converting enzyme inhibitors and ARBs have been shown to increase ACE2 activity in normotensive and hypertensive rats, particularly in the heart, kidneys, and myocardial infarct areas (47, 53). Moreover, ACE inhibitors enhance insulin sensitivity by increasing serum bradykinin levels (101).

In obese mice, ACE decreases while ACE2 increases following exercise, which may explain the heightened severity and mortality rates of SARS-CoV-2 infection in obese individuals due to increased viral loads (102, 103). In obese Zucker rats, increased salt intake reduces ACE2 expression in the renal cortex of damaged kidneys (43, 44). Similarly, at unilateral nephrectomy, biopsies from the remaining kidney show an increased ACE/ACE2 mRNA and protein ratio, a trend also observed in immunoglobulin A nephropathy and diabetic nephropathy (43, 44). These findings align with the poor prognosis seen in renal and diabetic patients with SARS-CoV-2 infection (51). Reduced Ang(1-7) production associated with this imbalance may exacerbate disease severity in such patients (103). Ang(1-7) exerts multiple beneficial effects through its interaction with the Mas receptor, including activation of mitogen-activated protein kinase (MAPK), NADPH oxidase, TGF-β1, EGF, and NFκB pathways (6, 45). In contrast, Ang2 has trophic, proliferative, and prothrombotic effects (43), whereas Ang(1-7) exhibits cardioprotective (1, 45), anti-proliferative, and anti-growth effects on vascular smooth muscle cells, cardiac myocytes, fibroblasts, and renal cells, such as glomerular and proximal tubule cells (1, 51).

Dysfunction of the Mas receptor gene (Mas1) in animal models leads to cardiovascular and renal abnormalities, including myocardial dysfunction, cardiac fibrosis, hypertension, endothelial dysfunction, renal fibrosis, glomerular dysfunction, insulin resistance, and dyslipidaemia (6, 44). Ang(1-7), through the Mas receptor, promotes systemic and regional vasodilation, diuresis, and natriuresis (43, 104).

In humans, Ang(1-7) has a half-life of approximately 0.5 h (43, 45). It is rapidly detectable in the bloodstream after injection, reaching peak plasma concentration within 1 h (43). Ang(1-7) is primarily metabolized in the lungs by ACE and can also be degraded by aminopeptidase and neprilysin (4, 43).

Maintaining a balance between Ang2 and Ang(1-7) is essential for ensuring proper perfusion (67, 105). At high doses, Ang(1-7) can induce tachycardia and arrhythmias due to extensive vasodilation (43, 105). However, localized and controlled external application of Ang(1-7) in low concentrations, aligned with its pharmacokinetics and pharmacodynamics, can interfere with the pathophysiological mechanisms driving ARDS, cardiac pathologies, kidney dysfunction, cytokine storms, and other complications that contribute to SARS-CoV-2-associated mortality (1, 105). Theoretically, this approach may prevent poor prognoses in organ failures and pathologies associated with SARS-CoV-2 infection (45).

Most of the primary pharmacological tools used to study the ACE2/Ang(1-7)/Mas receptor axis are Mas agonists that stimulate nitric oxide production and release (6, 47). These include Ang(1-7), AVE0991, CGEN861, CGEN856, and cyclic Ang(1-7) derivatives such as CGEN856S (43, 47). Among these, AVE0991 exhibits high affinity and specificity for Mas receptors, with minimal affinity for AT1 and AT2 receptors (43, 47). Ang(1-7) and AVE0991 compete for binding to Mas receptors (6, 67), with the IC50 value of AVE0991 reported to be approximately 1/108 mol/L (6).

Studies with TXA-127 or TRV-027, synthetic derivatives of Ang(1-7) showed that they are not effective (106). Another study was conducted with the recombinantly produced derivative of Ang(1-7). In this study, it was found that recombinant Ang(1-7) was well tolerated by patients with pneumonia due to COVID-19 requiring intensive care with oxygen saturation below 90%. In the same study, when Ang(1-7) was given by infusion, the number of days requiring oxygen and the length of stay in intensive care decreased in all patients. However, when they evaluated the patients who received Ang(1-7) infusion in Phase 2, they stated that it was not effective. We can think that Ang(1-7) may be more effective before the development of pneumonia related to COVID-19 or in a situation where there is a functional lung reserve that will respond to Ang(1-7) (107).

#### 3.4.2 Role of ACE2 and Ang(1-7) in long COVID [post-acute COVID-19 syndrome (PACS)]

“Long COVID syndrome”, as defined by the United States Centers for Disease Control and Prevention, involves the persistence of symptoms and signs for more than 4 weeks after SARS-CoV-2 infection (36, 37). The WHO defines it as symptoms and signs that last for more than 3 months after infection (36, 37). The primary symptoms of PACS include chronic fatigue syndrome, respiratory distress, reduced exercise tolerance, chest pain, postural orthostatic tachycardia, dysautonomia, and thrombotic complications (37, 41). PACS has been shown to occur in 10% of SARS-CoV-2 cases (36). It was determined that 12.6% of patients with PACS were asymptomatic at the initial detection of SARS-CoV-2 (38). Additionally, 76% of PACS patients required hospitalization (37). An examination of the sera from 31 PACS patients revealed the presence of two to seven types of functional autoantibodies against G-protein-coupled receptors (GPCR-fAABs) (6, 38). In contrast, these autoantibodies were detected only in a small group within healthy control studies. Ang(1-7) signals via a GPCR in the mitochondrial transmembrane (38).

It has been found that these fAABs exhibit no positive or negative chronotropic effects when interacting with the B2 adrenoceptor and M2 muscarinic receptor. In the same study, 29 of these 31 patients (90%) developed autoantibodies against the AT1 receptor, with which Ang2 interacts, and the MAS receptor, which interacts with Ang(1-7) (38). Notably, autoantibodies against the AT1 receptor have also been previously identified in patients with malignant hypertension and kidney disease (38), as well as in kidney transplant patients experiencing renal rejection (38).

An article from the Veterans Affairs Department in the USA reports a significant increase in cardiovascular diseases, heart failure, arrhythmias, and fainting in over 150,000 individuals within 1 year of SARS-CoV-2 infection (41). It is claimed that at least 65 million people have experienced PACS, including those without severe COVID-19 symptoms, with numbers rising daily (36, 41).

Over 200 symptoms have been identified in PACS (41). PACS has also been reported in children and uninfected individuals who have received the SARS-CoV-2 vaccine for prophylaxis (38, 41). The rate of vaccine-induced PACS among all PACS patients is reported to be between 10% and 12%. The incidence of PACS is 10%–30% in patients who were not hospitalized and between 50% and 70% in those who were hospitalized (41).

#### 3.4.3 Adverse effects of spike-based COVID-19 vaccines

The symptoms in COVID-19 patients show similarities with the adverse drug reactions (ADRs) reported with spike-based COVID-19 vaccinations. Therefore, binding of the spike subunit S1 of SARS-CoV-2 viruses and vaccine spikes to the host receptor enzyme ACE2 may be responsible for the same results. This supports the conclusions of the main mechanism of action of spike-based COVID-19 vaccines, namely, downregulation of ACE2 by the spikes (108).

It is highly possible that S1 of vaccines may increase adverse outcomes by causing long-term ACE2 dysfunction (109). It also appears that ADRs of spike-based COVID-19 vaccines are much more diverse and frequent than ADRs of previous vaccines (109). Signs and symptoms detected in PACS are also similar to ADRs after vaccination (41). For this reason, the 10%–12% rate of the development of PACS after vaccination is significant (41).

The European Medicines Agency (EMA) collects all side effects reported by authorities of European countries. Analysis of these reports revealed a considerable number of side effects, some of which have life-threatening consequences. These include acute cardiovascular reactions, blood clotting disorders, embolisms, thrombosis, myocarditis, vasculitis, and disorders affecting the nervous, musculoskeletal, skin, and intestinal systems, as well as various autoimmune or inflammatory diseases (109, 110).

## 4 Conclusion and future perspectives

The role of the RAAS system in acute, post-acute and long-term pathologies detected in SARS-CoV-2 infection has not been fully clarified in previous studies. This study aims to see the whole picture by synthesizing the intersecting findings in different studies on the role of RAAS in pathology. This study is and should be a narrative synthesis. In this way, the whole mechanism can be seen and the hypothesis steps and connections can be shown. In this way, it will be possible to produce solutions for SARS-CoV-2 treatment and prevention of complications, and to develop new treatment and protection agents. This study will be a roadmap for studies confirming these hypothesis steps and connections with cross-sectional experimental research.

The interaction with RAAS elements is not taken into account in current vaccine designs. It is confirmed that the S1 protein on the surface of SARS-CoV-2 enters the cell by endocytosis by interacting with ACE2 on the cell surface. In this case, the substrate of ACE2, Ang 2, and S1 should have similar epitopes. For ACE2, Ang1 is a substrate, just like Ang2. In this case, since Ang2 is produced from Ang1 with ACE, it is likely that Ang1 has epitopes similar to S1. In this case, since S1 and ACE interaction are also possible, Ang2 production may also decrease. The decrease in Ang2 will increase ACE expiration with negative feedback to increase its production. This is a strong hypothesis that needs to be confirmed with *in vitro* and *in vivo* experimental studies.

In some studies, autoantibodies have been detected in complications after vaccination and COVID-19. Apart from the autoantibodies formed against Ang2, which are formed due to the similarity of S1 of SARS-CoV-2 to Ang2, there is also an important contribution of autoantibodies against different cell and tissue components of the extreme destruction of cells and tissues due to hypoxia related to perfusion disorder. The immune reaction formed against these tissues and cell components contributes to the situation called cytokine storm, which is important in the prognosis of SARS-CoV-2 and is formed by the extreme formation of cytokines in signal transmission. It does not appear to be a condition due to cell and tissue damage specific to the lungs alone.

In studies after PACS, a limited number of autoantibodies have been screened and the connections have not been clearly established. Autoantibodies have been detected against vascular cell components. However, considering the hypothesis of this study, it is possible that autoantibodies will be detected against many tissue and cell components other than these. It has also been reported that a significant number of PACS cases occur after vaccination without SARS-CoV-2 infection. Autoantibodies are also detected in this group. This situation necessitates questioning the relationship between S1 and ACE2 in all current vaccines.

**FIGURE 3:**
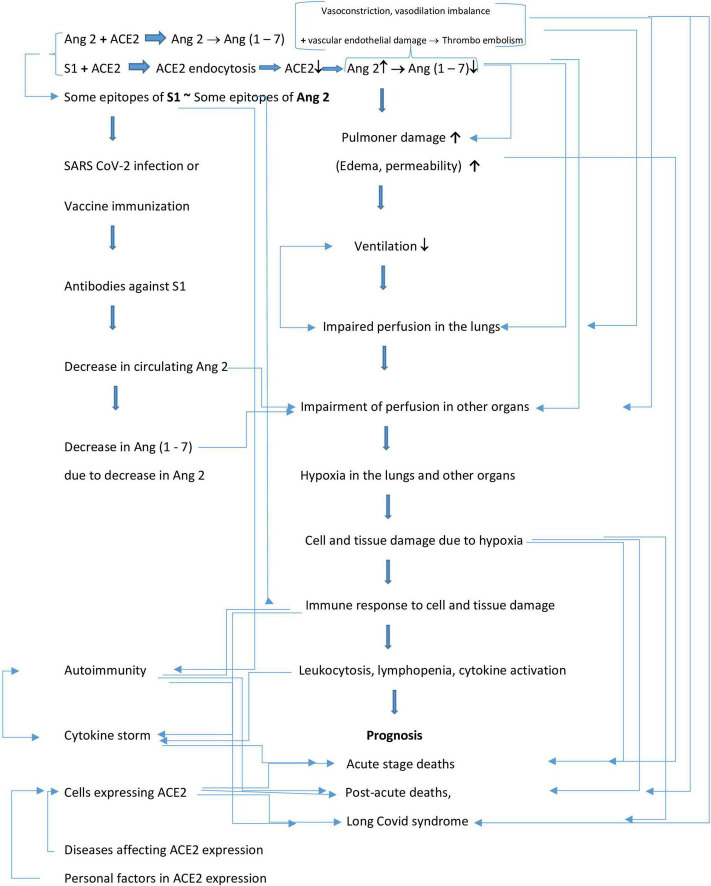
Hypothesis synthesis diagram. Figure 3 reference (1–110).

The pathological research findings after SARS-CoV-2 infection, the nature of the complications and symptoms, and the relationship between the deterioration in RAAS function are theoretically very clear. It is scientifically necessary to design experimental studies and confirm the hypothesis to clarify this situation. However, the studies are limited and the connections have not been clearly established. Therefore, multidisciplinary data could be hypothesized as a whole. The hypothesis flowchart is schematized in Figure 3.

In addition, according to the post mortal pathological research findings, the relationship with the deterioration in RAAS function are extremely clear after SARS-CoV-2 infection. Numerous studies have reported that ARDS is the most prevalent complication leading to mortality in the pathogenesis of SARS-CoV-2. ACE2 is associated with ARDS as well, and SARS-CoV-2 proliferates by infecting ACE2-expressing cells in other organs and tissues. Research also suggests that the virus directly damages Type 2 pneumocytes in the lungs, and that cytokine storms contribute to ARDS. However, SARS-CoV-2 pathology cannot be solely explained by this mechanism.

It is understood that SARS-CoV-2 spreads through the blood to secondary organs following initial viremia in the lungs. In virus-infected cells, ACE2 is internalized through endocytosis, resulting in a reduction of ACE2. In this context, administering Ang(1-7) via inhalation in low doses can deliver it directly to the respiratory tract and alveoli. The other option is intravenous administration of low-dose Ang(1-7), which will route it through the venous system to the right atrium, right ventricle, and lungs via the pulmonary artery, localizing its effects and potentially reducing hydrostatic pressure in the pulmonary capillaries. This could prevent fluid retention in the lungs by promoting cell and tissue integrity and reducing permeability. Additionally, thromboembolic events can be reduced with the protective, anti-fibrotic, anti-thrombotic and anti-proliferative properties of Ang(1-7).

It is suggested that the most physiological treatment option to prevent complications in SARS-CoV-2 infections can be the ACE2 derivative Ang(1-7). A summary of the effects of Ang(1-7) administration is as follows:

(1) Decreases the local demand for Ang(1-7) at the initial replication site.
(2) Reduces the need for ACE2 function.
(3) Lowers ACE2 production.
(4) Reduces SARS-CoV-2 attachment, entry, and proliferation by decreasing ACE2.
(5) Utilizes the protective, anti-fibrotic, antithrombotic, and anti-proliferative properties of Ang(1-7).
(6) Reduces the utilization of Ang2 as a substrate for ACE2.
(7) Facilitates Ang2-AT1 function necessary for perfusion by increasing Ang2.
(8) Improves ventilation and perfusion in the lungs, thus reducing hypoxia.
(9) Minimizes cytokine release and cell death due to reduced hypoxia in tissues.
(10) Decreases autoimmune events induced by tissue destruction, thereby reducing long COVID syndromes and symptoms.
(11) Minimizes lung pathology, whereby the patient’s immune system can focus its efforts on combating the virus within a limited area, rather than contending with a cascade of pathologies that eventually spread to other organ systems.

Unfortunately, the SARS-CoV-2 pandemic has shown that the Fordist structure negatively impacts our ability to find effective solutions and manage the pandemic. This problem in the current scientific structure stems from the fact that the concept of medicine has been designed by dividing it into sections, considering previous requirements. In order to serve the society with the increase in knowledge in medicine, we divide medical knowledge into branches, and this division into branches intensifies us by focusing on a perspective. Although we have areas of knowledge that intersect with different perspectives, the pathologies we see and the templates we produce for them limit our ability to see and evaluate from different perspectives. There are symptoms and findings in SARS-CoV-2 infection that are of interest to many branches and that need to be solved. When each branch evaluates from its own perspective, there may be a problem in seeing the big picture and synthesizing information and reaching an effective, uncomplicated solution.

Therefore, given that epidemics are inevitable and ongoing, we need to devise new strategies and perspectives, as our current approaches are insufficient. This study leverages insights from the SARS-CoV-2 pandemic for managing both this and future pandemics. Our observations highlight the necessity of multidisciplinary research and thinking, which we advocate for this review.

## Data Availability

The original contributions presented in this study are included in this article/supplementary material, further inquiries can be directed to the corresponding author.
